# Design and Study of a Microfluidic Chip for Two-Stage Sorting of Oil Wear Debris Based on Magnetophoretic

**DOI:** 10.3390/mi17040397

**Published:** 2026-03-25

**Authors:** Zhiwei Xu, Hongpeng Zhang, Haotian Shi, Wenbo Han, Bo Liu

**Affiliations:** 1 Marine Engineering College, Dalian Maritime University, Dalian 116026, China; xuzhiwei0053@dlmu.edu.cn (Z.X.); wenbo_han@126.com (W.H.); 13674137039@163.com (B.L.); 2 State Key Laboratory of Maritime Technology and Safety, Dalian 116026, China; 3 College of Safety Science and Engineering, Nanjing University of Science and Technology, Nanjing 210094, China; dmu6hao@163.com

**Keywords:** debris sorting, magnetophoresis, two-stage sorting

## Abstract

Oil analysis is one of the main means to obtain the working status of important friction pairs in ship and Marine engineering equipment at present. Analyzing the wear mechanism by analyzing the particle size, morphology, properties and other characteristics of metal abrasive particles in the oil is an important basis for achieving health monitoring and scientific maintenance of ship and Marine engineering equipment. Classifying the abrasive particles in the oil according to their particle size is an important step in sample pretreatment. This paper proposes a two-stage sorting microfluidic chip for wear debris based on magnetophoresis. By setting up external permanent magnets in a stepwise manner in the primary and secondary sorting areas, gradient magnetic fields of different magnitudes were formed. The effects of different sample flow rates, sheath fluid flow rates and sheath flow ratios on the pre-focusing before sorting and the sorting effect were studied. The primary sorting of ferromagnetic metal wear particles larger than 50 µm and the secondary sorting of those smaller than 50 µm have been achieved. The primary sorting can serve as an early warning for abnormal equipment wear, while the secondary sorting can provide data support for the scientific formulation of maintenance plans based on equipment requirements. This work provides a new idea and method for the rapid determination of lubricating oil contamination in engineering equipment.

## 1. Introduction

Abnormal wear of friction pairs is one of the main causes of mechanical equipment failure [1]. Oil is a key medium for monitoring the operating status of equipment. Indicators such as oil viscosity, physical and chemical properties and contamination level can reflect the degree of equipment wear to a certain extent [2]. By detecting and analyzing metal wear debris in oil, mechanical equipment status warning and fault diagnosis can be achieved [3]. In recent years, various online metal wear debris detection sensors and related technologies have begun to flourish [4]. Among them, the mainstream ones are optical [5], acoustic [6], magnetic plug-in [7], inductive [8], capacitive [9] and other online metal wear debris monitoring sensors. Ren et al. proposed an inductive metal wear debris detection sensor [10]. The sensor consists of an external excitation coil and multiple internal sensing coils. Different from the traditional structure, the sensor improves the sensitivity by increasing the ratio of the volume of abrasive debris to the sensing area. A double semicircular sensor was designed and manufactured for preliminary experimental verification. The results show that 120 μm ferromagnetic abrasive debris and 210 μm non-ferromagnetic abrasive debris can be effectively detected in a 34 mm pipe. In order to increase the detection flux of the sensor, Zhu et al. designed a multi-channel abrasive debris detection sensor [11]. Time division multiplexing technology is used in the sensor signal processing link to realize the function of collecting multi-channel responses by a group of measurement units. At the same time, each induction coil adopts an L-C-R resonant structure to ensure that the flux is increased while maintaining a high detection sensitivity. The experimental results show that the sensor can effectively detect 50 μm wear debris at a flux of 460 mL/min. However, the above methods and research have problems such as complex circuits and structures, low detection accuracy and difficulty in miniaturization. Moreover, refined oil analysis still needs to be carried out in the laboratory [12], such as ferrography [13], spectral analysis [14] and optical particle counting. Especially in the research of wear mechanism, ferrography analysis and microspot analysis have obvious advantages. Classifying metal abrasive particles by particle size remains an important pretreatment step in spectroscopy and smear preparation and is the key to improving the analysis efficiency. In recent years, microfluidic technology has developed rapidly, and various methods for manipulating micro- and nano-scale particles in microchannels have been greatly developed, including optics [15], electrophoresis/dielectrophoresis [16], acoustics [17] and magnetism [18]. Related research has been widely used in the fields of biomedicine, environmental monitoring, chemical analysis, etc., and the main forms include micromixers, microsorting, and microreactors [19,20,21,22,23,24]. Among them, magnetophoresis microsorting technology has shown unique advantages in the induction and manipulation of metal wear debris in fluids.

Magnetophoresis sorting generates a high-intensity high-gradient magnetic field in the sorting area inside the chip by setting up an external magnetic source. Due to the different magnetic forces on the particles in the magnetic field, they will produce different degrees of deflection in the direction perpendicular to the channel [25]. The exit angles in different directions are set along the particle trajectory to achieve effective sorting and detection of different particles [26]. Zhou et al. used soft lithography technology to make micro-magnets of different shapes on a microfluidic chip [27] and studied the differences in the gradient magnetic fields generated by magnets of different shapes in the sorting area. The simulation results show that the magnetic field gradient generated by rectangular micro-magnets is higher than that of semicircular and triangular micro-magnets. This also suggests that rectangular micro-magnets have higher precision when performing manipulation and sorting. Yeast cell separation experiments were carried out, which once again confirmed the accuracy of the research results. In terms of improving sorting efficiency, Guidice et al. improved the rectangular micro-magnet [28]. A particle manipulation and sorting device with pre-focusing function was designed. The device consists of two modules in series. The front end of the straight channel is used for particle focusing, and the rear end is within the range of the external magnetic source. A gradient magnetic field area is formed in the channel to achieve particle sorting. Experiments have shown that the design of the pre-focusing module increases the sorting efficiency from 80% to 92%. These studies have laid a solid foundation for the design optimization and expansion of application scenarios of magnetophoretic microfluidics. However, so far, research related to magnetophoretic microfluidic manipulation and sorting has mainly focused on the field of biochemistry, and there are few reports on the sorting of metal wear debris in lubricating oil [29,30,31,32].

This study proposed a two-stage sorting microfluidic chip for oil abrasive debris based on magnetophoresis. It further expanded the application of magnetophoresis microfluidics in the field of mechanical manufacturing and provided a new methodological approach for the rapid analysis and detection of oil pollution. By setting up two-level differential magnetic fields, the primary sorting of large and small wear debris and the secondary fine separation of small wear debris were achieved. The setting of the particle size threshold is consistent with the range of abrasive particle sizes that are of primary concern in oil analysis in industry. The main research focused on the migration behavior of high magnetic permeability particles in high-viscosity media and discussed the influence of the magnetic field on the movement trajectory of ferromagnetic metal abrasive particles in the oil, that is, the relationship between the distance between the external magnetic source and the channel wall and the magnetic field distribution in the separation area. Secondly, the effects of the flow velocity of the oil sample and the flow velocity ratio of the sheath liquid sample on the particle separation efficiency were analyzed, and the separation effect of the chip was verified through experiments.

## 2. Theoretical

To analyze the force on the metal abrasive debris in the oil during the sorting process, they are mainly subjected to the trailing force F_d_ of the oil flow as well as the magnetophoretic force F_m_. Due to the low Reynolds coefficient of the oil in the microchannel, other forces such as inertial lift on the abrasive debris are negligible. Where the trailing force Fd is in the direction of the channel axis, the magnetophoretic force Fm is along the radial direction of the channel, which is a key factor in the deflection of the debris during the sorting process. For an abrasive debris in a non-uniform gradient magnetic field, the magnetophoretic force on it can be expressed as

(1) $$F_{m}=\frac{V_{p}\left(\chi_{p}-\chi_{f}\right)}{\mu_{0}}\left(B\cdot \nabla \right)B$$

where V_p_ is the volume of the abrasive debris, *χ*_p_ is the magnetization of the abrasive debris, *χ*_f_ is the magnetization of the fluid medium, *μ*_0_ is the vacuum permeability and ***B*** is the magnetic induction strength. From the equation, it can be seen that the magnitude of *χ*_p_ and *χ*_f_ is the key to determine whether the magnetophoretic force is positive or negative. When *χ*_p_ is larger than *χ*_p_, the particles in the channel are subjected to a positive magnetophoretic force; on the contrary, they are subjected to a negative magnetophoretic force. And the size of the force subjected to magnetophoresis is related to the volume size of the abrasive debris, which is also the theoretical basis for us to utilize magnetophoresis for the sorting of abrasive debris of different sizes.

Furthermore, the trailing force of the fluid flow on the abrasive debris can be expressed as

(2) $$F_{d}=3\pi \eta_{f}D_{p}f_{D}\left(u_{f}-u_{p}\right)$$

where *η*_f_ is the dynamic viscosity of the fluid medium, D_p_ is the diameter of the abrasive debris, ***u***_f_ and up denote the velocity of the fluid and the abrasive debris, respectively, and *f*_d_ represents the coefficient of resistance of the fluid medium to the abrasive debris, which can be obtained by calculating the following equation:

(3) $$f_{D}={\left[1-\frac{9}{16}\left(\frac{D_{p}}{D_{p}+2L}\right)+\frac{1}{8}{\left(\frac{D_{p}}{D_{p}+2L}\right)}^{3}-\frac{45}{256}{\left(\frac{D_{p}}{D_{p}+2L}\right)}^{4}-\frac{1}{16}{\left(\frac{D_{p}}{D_{p}+2L}\right)}^{5}\right]}^{-1}$$

where *L* is the shortest distance from the abrasive debris surface to the channel wall. Then, the kinetic equation of the abrasive debris in the channel can be expressed by the following equation:

(4) $$m_{p}\frac{du_{p}}{dt}=F_{m}+F_{d}$$

## 3. Modeling and Design

**Figure 1:**
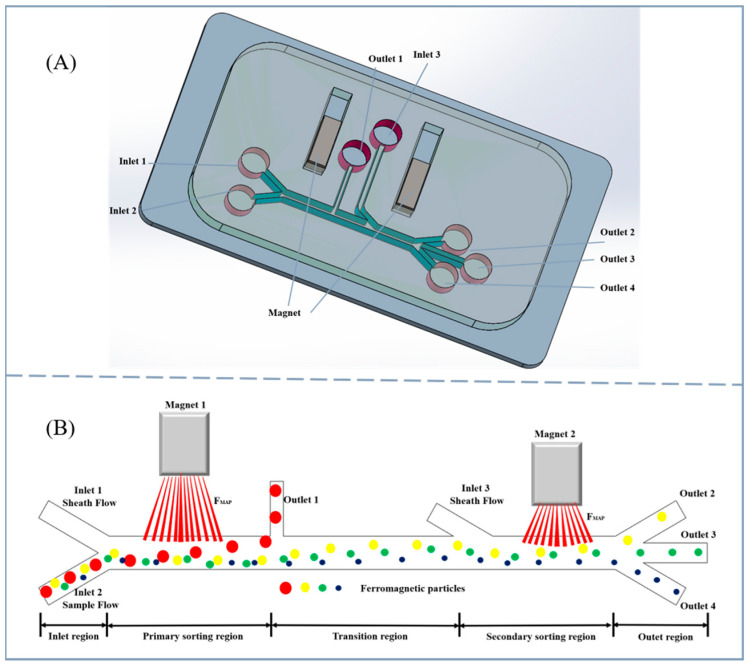
(**A**) Three-dimensional structure of the chip; (**B**) schematic diagram of chip size and sorting.

The two-stage oil–liquid wear debris sorting microfluidic chip designed in this study is shown in Figure 1. Figure 1A is a three-dimensional structure diagram of the chip. Figure 1B describes in detail the main relative positions and dimensions of the chip. The chip consists of 3 inlets and 4 outlets, of which inlets 1 and 3 are sheath liquid inlets and inlet 2 is the sample inlet. The sample is oil mixed with ferromagnetic metal wear debris, the density of the oil is 846.2 kg/m^3^, the dynamic viscosity is 0.02 Pa·s and the relative magnetic permeability of the wear debris is 300 (oil used in this experiment was the Changcheng lubricant of WO-20 grade produced by Sinopec Lubricants Co., Ltd.). Outlet 1 is the primary sorting outlet, and outlets 2, 3 and 4 are the secondary sorting outlets. The sorting area consists of two parts, including the primary sorting area at the front end and the secondary sorting area at the rear end. The width of the inlet and outlet channels is 300 μm and the length is 1000 μm. The width of the main channel in the sorting area is 500 μm and the length is 20,000 μm. Under the action of the permanent magnet placed on one side of the chip channel, a local gradient magnetic field is formed in the channel. Abrasive particles of different particle sizes undergo different degrees of lateral deflection under the action of magnetophoretic force in the gradient magnetic field, thereby achieving the sorting of abrasive particles of different particle sizes (iron particles were produced by Huiji Metal Materials Co., Ltd.).

This work aims to make the metal wear debris in the oil undergo positive magnetophoresis in the chip channel by designing appropriate permanent magnet spacing, sample and sheath fluid flow rates and soft magnetic material structure dimensions. Wear debris of different particle sizes are subjected to different magnetophoretic forces, resulting in different lateral displacements, and then flow out from different outlets, thereby achieving effective sorting of wear debris of different particle sizes.

This paper uses COMSOL Multiphysics (v6.3) for simulation analysis and applies three modules: magnetic field, no current, laminar flow and fluid flow particle tracking. The three modules are used to analyze and calculate the magnetic field distribution, flow field distribution and the influence of flow field and magnetic field coupling on the motion trajectory of wear debris in the chip channel. The magnetic field no-current module uses Gauss’s magnetic law and Maxwell–Ampere’s law as the control equation. The laminar flow module uses the Navier–Stokes equation as the control equation.

### 3.1. A. Laminar Flow Model

In the microfluidic system, the Reynolds coefficient of the laminar flow is small, so its inertia term can be neglected, and the viscosity term of the Navier–Stokes equations plays a dominant role, and the Navier–Stokes and continuity equations controlling the flow field are

(5) $$\rho \left(u\cdot \nabla \right)u=\nabla \cdot \left[-pI+K\right]+F$$

(6) ρ∇·u=0

The corresponding boundary conditions are set as follows: no slip in the channel wall, the inlet velocity is the normal inflow velocity, the magnitude of which can be adjusted according to the demand, and the boundary control condition of the outlet is the pressure, and the outlet pressure is set to be consistent with the atmospheric pressure, i.e., P = 0.

### 3.2. B. Static Magnetic Field Model

Since the static magnetic field model is chosen to be a magnetic field with no current module, there is no free current, only the magnetization vector field, and the magnetic field strength is spinless, i.e., there is a magnetic scalar potential Vm. Thus, Maxwell–Ampere’s law and Gauss’s law, which control the magnetic field, are, respectively,

(7) $$H=-\nabla V_{m}$$

(8) ∇·B=0

The magnetization model for the magnet’s intrinsic relation B-H is set to the residual flux density, defined by the equation

(9) $$B=\mu_{0}\mu_{\mathrm{rec}}H+B_{r},\;B_{r}=\left\|B_{r}\right\|\frac{e}{\left\|e\right\|}$$

where Br denotes the residual flux density, e denotes the direction, μrec denotes the remanent permeability and μ0 is the vacuum permeability. Furthermore, on the outermost boundary of the entire computational domain (the air domain), a magnetic insulation boundary condition was imposed. The mathematical expression of this boundary condition is **n** × **B** = 0.

In addition to this, the controlling equation for the motion of the particle in the particle tracking model is Newton’s second law. The location of the boundary conditions is mainly the channel wall surface. Here, the wall condition is set as “adhesion”.

Regarding the verification of grid independence, the simulation area in this study is a 25 mm × 50 mm region, which contains 5540 triangular grid cells. When using a finer grid, the simulation results do not show any significant changes, indicating that these results are very close to the exact solution of the governing equations.

## 4. Results and Analysis

**Figure 2:**
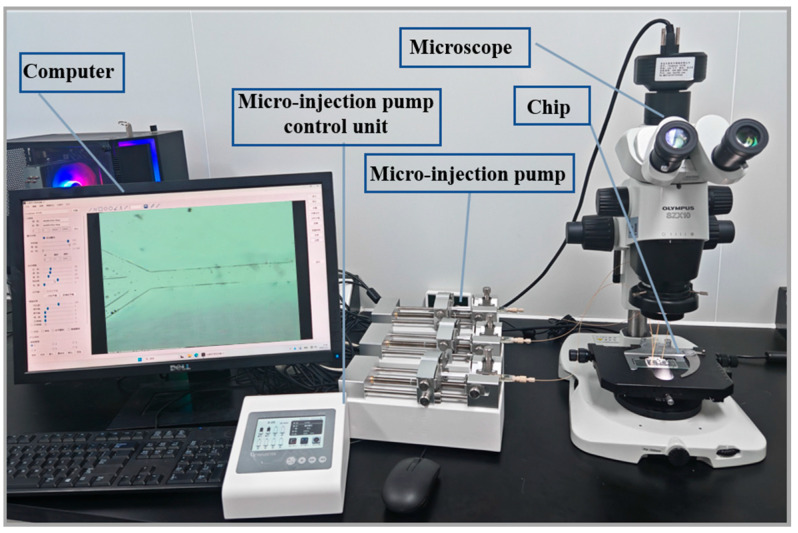
Experimental system.

To test the sorting effect of the chip, an experimental system was set up. As shown in Figure 2, the system includes a micro-injection pump (DK SPM03, D K INFUSETEK Co., Ltd.), a microscope (OLYMPUS SZX10, Japanese company Olympus, Tokyo, Japan), a computer and the microfluidic sensor chip we fabricated. Among them, the micro-injection pump is used to regulate the speed and flow rate of the oil passing through the sensor; a CCD (longbase 1610E, Qingdao Changji Medical Devices Co., Ltd., Qingdao, China) is installed on the microscope, which can display in real time the sorting situation of abrasive particles in the chip on the display screen. In addition, two concentrations of the test samples, namely, 10 mg/mL and 0.1 mg/mL, were prepared. They were used to complete the two aspects of the experiment, namely, the flow line distribution and the grinding particle separation. The sample with a higher concentration can better observe the flow line distribution situation, while the sample with a lower concentration was used for the experimental part of grinding particle separation. The primary sorting section and the secondary sorting section of the sorting chip were studied separately. First, the primary sorting of the sorter was investigated.

### 4.1. A. Primary Sorting

In the primary sorting section, the first thing to be explored is the impact of flow rate on the sorting efficiency of the sorter, which mainly includes the influence of the sample flow rate and the ratio of the sheath fluid to the sample flow rate. The sample flow rate was studied. The sample flow rates were set at 500 µm/s, 1000 µm/s and 1500 µm/s, respectively. The ratio of the sheath fluid to the sample flow rate was maintained at 1:1. Then, 40 µm and 55 µm abrasive particles were selected for simulation analysis.

**Figure 3:**
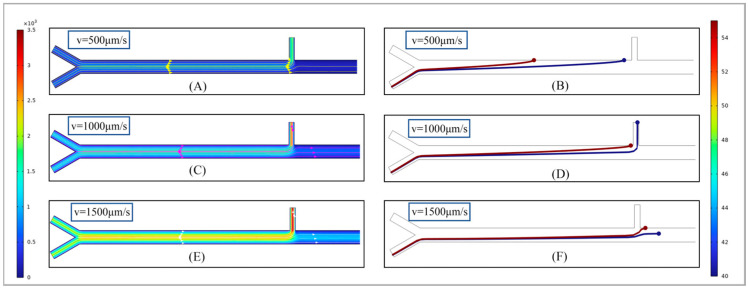
The influence of different sample flow rates on the sorting effect. (**A**,**C**,**E**) represent speed simulations of 500 µm/s, 1000 µm/s and 1500 µm/s, respectively; (**B**,**D**,**F**), respectively, represent the particle movement trajectories of 40 µm and 55 µm at flow rates of 500 µm/s, 1000 µm/s and 1500 µm/s.

As shown in Figure 3, there are velocity diagrams and abrasive particle movement trajectories within the three different sample flow rate sorting channels. From Figure 3A,C,E, it can be seen that as the sample flow rate increases, the velocity in the main channel significantly increases, and the velocity is the highest at the central position of the channel, indicating that as the sample flow rate increases, the central streamline of the fluid is located at the center of the channel. However, the movement trajectories of the abrasive particles under the corresponding flow velocity show significant changes. When the flow velocity is low, the time it takes for the abrasive particles to pass through the sorting area increases, the time during which they are affected by the magnetic swimming force also increases and the longitudinal displacement generated in the channel becomes larger. As shown in Figure 3B, the abrasive particles were adsorbed on the channel wall before reaching outlet 1 and thus failed to achieve effective separation. On the contrary, when the sample flow rate increased to 1500 µm/s, the time for the particles to pass through the separation area shortened, and the effect of the magnetic swimming force on the abrasive particles weakened. As a result, no effective separation occurred when the abrasive particles passed through outlet 1. Therefore, during the sorting operation, the sample flow rate should be maintained at a reasonable level to ensure the best sorting results.

**Figure 4:**
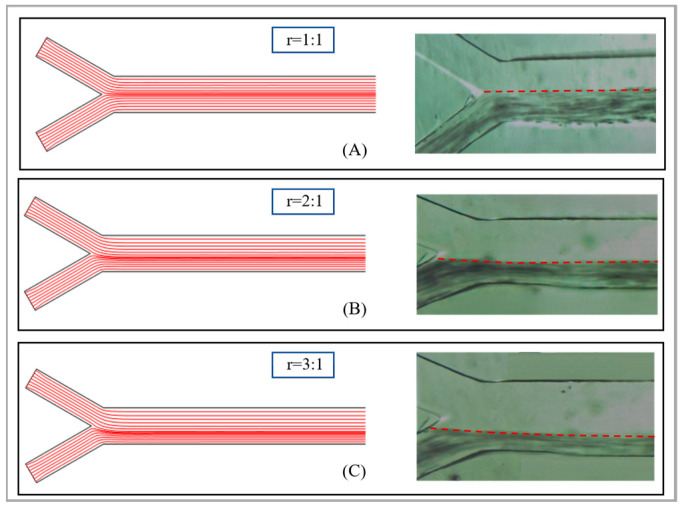
Flow streamline diagrams of fluids under different ratios of sheath fluid to sample flow rate. (**A**) 1:1; (**B**) 2:1; (**C**) 3:1. The dotted line here indicates that during the experiment, the centerline of the fluid also gradually moved downward as the flow velocity ratio increased, which is consistent with the simulation results.

Subsequently, this study investigated the influence of the ratio of the sheath fluid to the sample flow rate on the sorting effect. Considering that as the flow rate ratio increases, the total flow rate within the channel will also increase rapidly, we have determined the sample flow rate to be 500 µm/s. We will discuss and analyze three scenarios with ratios of 1:1, 2:1 and 3:1. Specifically, the sheath fluid flow rate in the primary sorting area is set to 500 µm/s, 1000 µm/s and 1500 µm/s. The simulation and experimental analysis were conducted using 40 µm and 55 µm abrasive particles. As shown in Figure 4, it is a flow line diagram of the fluid within the sorting channel. It can be seen that as the flow velocity ratio increases, the centerline of the fluid begins to gradually move downward from the central position. Correspondingly, when the abrasive particles pass through the sorting area, the magnetic swimming force also decreases. The force acting on the small particle diameters decreases, thereby achieving the separation of abrasive particles of different particle sizes.

**Figure 5:**
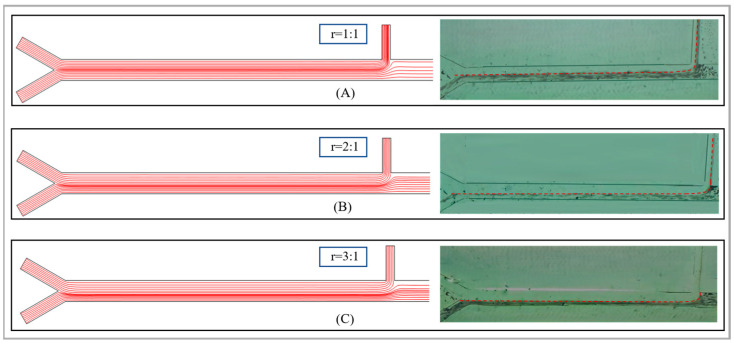
Particle motion trajectories in the primary sorting region under different flow velocity ratios. (**A**) 1:1; (**B**) 2:1; (**C**) 3:1. The red dotted line represents the movement trajectory of the particles. It can be observed that as the flow velocity ratio increases, the abrasive particles will not exit from outlet 1 in the primary sorting area, but will all enter the secondary sorting area.

As shown in Figure 5, it depicts the particle movement trajectories in the primary sorting area under different flow velocity ratios. Moreover, both the simulation and the experiment in the figure were conducted without the addition of an external magnetic field. As can be seen from the figure, when the flow ratio is 1:1, some of the abrasive particles will flow out from outlet 1 when they pass through it. The particle sizes of the particles that flow out are not uniform and thus the size sorting of the abrasive particles cannot be achieved. As the flow rate ratio increases to 2:1, from the simulation diagram of the fluid flow lines, it can be observed that when the fluid centerline passes through outlet 1, it will basically continue to flow completely in the rear direction without any obvious separation flow phenomenon. Moreover, the experimental results also show that at this flow rate ratio, only a small number of abrasive particles will flow out from this outlet when passing through it. If the flow velocity ratio is further increased to 3:1, it can be observed that when the centerline of the fluid passes through outlet 1, it will completely flow backward, and the abrasive particles will also flow completely backward. Therefore, during the sorting operation, the ratio of the sheath fluid flow rate to the sample flow rate should be at least 1:2 or higher to ensure that all abrasive particles can all flow to the next area without an external magnetic field. Furthermore, by setting an external magnetic source, the effective sorting of abrasive particles can be achieved by utilizing the different magnetic propulsion forces exerted on particles of different sizes.

**Figure 6:**
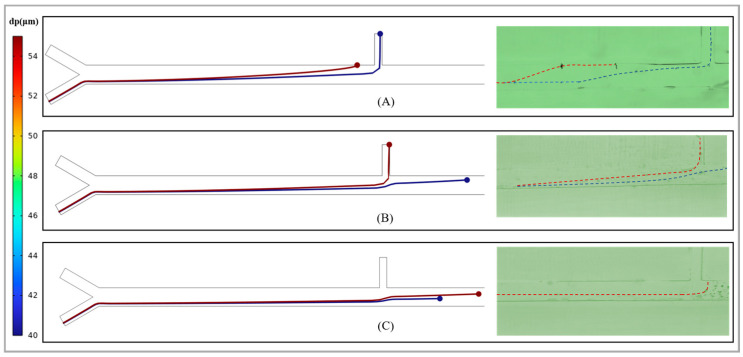
Particle motion trajectories in the primary sorting region under different flow velocity ratios. (**A**) 9000 µm; (**B**) 11,000 µm; (**C**) 12,500 µm. In (**A**) and (**B**), the red dotted lines represent the trajectory of 55 µm abrasive particles, while the blue dotted lines represent the trajectory of 40 µm abrasive particles. In (**C**), it shows that both the 55 µm and 40 µm abrasive particles have entered the secondary sorting area.

The distance between the external magnetic source and the channel is also one of the key factors determining the magnitude of the magnetic drag force that the abrasive particles experience. In this study, a N35 permanent magnet was placed on one side of the separator as an external magnetic source. The initial distance was set at 10,000 μm, which refers to the distance from the lower boundary of the magnet to the centerline of the main channel of the separator. The distance was continuously increased or decreased by 500 μm. During this process, numerical simulations were conducted on the movement trajectories of 40 µm and 55 µm abrasive particles, and the results were verified through corresponding experiments. The research results are shown in Figure 6. When the distance is 9000 µm, in the simulation diagram of the abrasive particle trajectory, the 40 µm abrasive particles flow out from outlet 1, while the 55 µm abrasive particles are adsorbed on the channel wall due to the excessive magnetic swimming force they encounter. The experimental phenomenon also verifies that effective separation of the abrasive particles cannot be achieved at this distance. When the distance reaches 11,000 µm, both the simulation results and the experimental results show that the abrasive particles of 55 µm and 40 µm flow towards outlet 1 and the secondary sorting area, respectively. When the distance increased by 12,500 µm, both the particles of different sizes flowed backward and reached the secondary sorting area. Therefore, in order to ensure effective separation in the primary sorting area, the distance between the N35 permanent magnet and the centerline of the main channel should be set at around 11,000 µm.

### 4.2. B. Secondary Sorting

**Figure 7:**
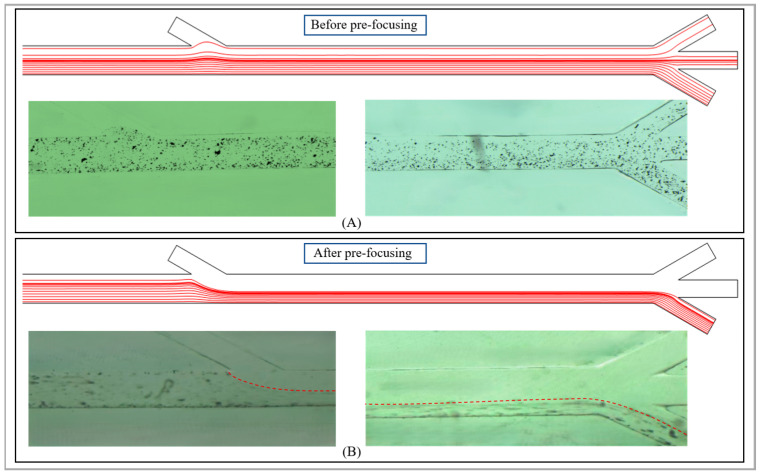
The influence of the velocity of the secondary sheath flow on the pre-focusing of sorting. (**A**) Before pre-focusing; (**B**) after pre-focusing. The red dotted lines in the figure represent the movement trajectories of the abrasive particles in the secondary sorting area. After the pre-focusing of the abrasive particles by the sheath flow, it can be seen that the trajectories of the particles become more orderly and all will flow out from channel 4. The experimental results are consistent with the simulation results.

When the abrasive particles pass through the transition zone and enter the secondary separation, as shown in Figure 7, when the sheath fluid flow rate at the inlet 3 is 0, it can be seen that both the simulation diagram of the streamline and the experimental results show that the movement of the abrasive particles is rather random and irregular. Therefore, it is necessary to set the sheath flow at inlet 3 at an appropriate speed to achieve the pre-focusing of the sample abrasive particles. The sheath flow setting should ensure that the abrasive particles can all flow out from outlet 4 without any external magnetic source. When an N35 magnet is added externally, the abrasive particles of different sizes in the channel will be subjected to different magnitudes of magnetic drag force. Especially for the larger-sized abrasive particles, they will undergo significant lateral displacement under the action of the magnetic drag force and eventually flow out from outlet 2 and outlet 3. Through continuous adjustments, it was finally determined as shown in the figure. When the sheath fluid flow rate at inlet 3 is above 400 µm/s, it can ensure that the flow lines in the main channel converge and flow out from outlet 4. As can be seen from the experimental graph, the abrasive particles have achieved pre-focusing and have mostly flowed out from outlet 4.

Next comes the setting of the external magnetic field in the secondary sorting area. Just like the first-level sorting, it is also necessary to make a reasonable adjustment to the distance between the external magnetic source permanent magnet N35 and the centerline of the main channel. Since the abrasive particles in the secondary sorting are smaller in size compared to those in the primary sorting, the distance between the permanent magnet and the main channel is also relatively closer. Overall, the two external magnets in the primary sorting and secondary sorting present a stepped distribution. The same method as in the primary sorting area is still used to ultimately determine the exact position of the external permanent magnet N35.

**Figure 8:**
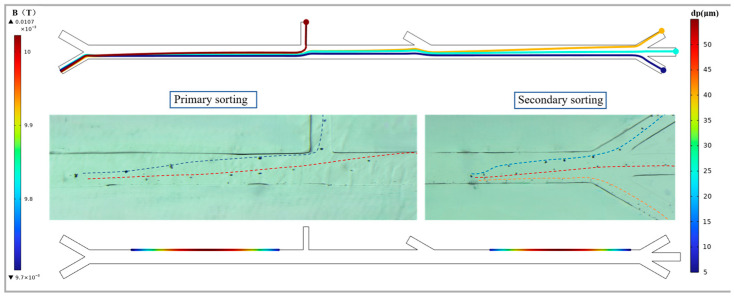
The magnetic field distribution and sorting effect of the chip at the channel wall surface. In the left figure, the abrasive particle trajectories in the primary sorting area are shown. The blue line represents the movement trajectory of the abrasive particles larger than 50 microns, while the red line represents the movement trajectory of the abrasive particles smaller than 50 microns. The figure on the right shows the grinding particle trajectories in the secondary sorting area. The blue color represents the trajectories of particles ranging from 30 to 50 microns, the red color represents the trajectories of particles ranging from 10 to 30 microns, and the orange color represents the trajectories of particles smaller than 10 microns.

However, the design of the differential selector is not merely a simple combination of individual modules and parameters; the coupling of the flow field and the magnetic field, as well as the interactions among particles, should all be fully taken into account. Based on the previous research, this work ultimately designed a microfluidic chip for two-stage separation of oil-based abrasive particles using magnetic levitation. The basic parameters are as follows: In the primary sorting area, the distance between the external magnetic source and the centerline of the channel is 10,500 µm, the sample flow rate at entrance 1 is 500 µm/s and the sheath fluid flow rate at entrance 2 is 1500 µm/s. In the secondary sorting area, the distance between the external magnetic source and the centerline of the channel is 10,000 µm, and the sheath fluid flow rate at entrance 3 is 700 µm/s. Using this chip, a sorting study was conducted on abrasive particles of 5 µm, 25 µm, 40 µm and 55 µm. The sorting situation of the abrasive particles is shown in Figure 8. Among them, the 55 µm sized abrasive particles flow out from outlet 1. After passing through the secondary sorting area, the 40 µm sized particles flow out from outlet 2, the 25 µm sized particles flow out from outlet 3 and the 5 µm sized particles flow out from outlet 4. At this point, by observing the distribution of magnetic field density on the channel wall in the sorting area, it can be seen that the magnetic flux density is the highest near the channel wall corresponding to the centerline of the magnet. The maximum magnetic flux density in the primary sorting area is 0.01 T, while that in the secondary sorting area is 0.0107 T. At the same time, the magnetic flux density at the strongest points on the channel walls of each sorting area was calculated along the direction perpendicular to the channel walls. The magnetic field gradient decreased gradually as the distance from the magnetic source increased. In the primary sorting area, the magnetic field gradient decreases from 0.45 T/m at the upper wall surface to 0.38 T/m at the lower wall surface. In the secondary sorting area, it drops from 0.55 T/m to 0.46 T/m.

Meanwhile, under these experimental parameters, we conducted an analysis and statistics on the microscope images of the particle sorting during the experiment. When the sample flow rate is set at 500 µm/s, based on the size of the sample inlet channel, it can be calculated that the flux of the oil sample processed by the differential separator is 0.9 µL/min. The sorting efficiency and purity of the differential selector are shown in the Table 1 below. In the primary sorting area, due to the interaction between particles and the insufficient gradient of the magnetic field, when sorting particles of 50 µm in size, some particles of around 40 µm will flow out from outlet 1. Similarly, there are problems at outlets 2 and 3. However, at outlet 4, since particles smaller than 10 µm are less affected by the magnetic force, their sorting efficiency and purity are relatively higher. Furthermore, due to the high density of the iron particles, the phenomenon of particle sedimentation during the sorting process has a significant impact on the sorting results. Therefore, the recovery rate of this sorter is only 91.73%.

## 5. Discussion and Conclusions

In this study, the two-level sorting of the chips not only enables early warning of equipment failures but also allows for in-depth research on the wear patterns of the equipment. The primary sorting area of the chip can sort and detect particles larger than 50 microns. When particles with larger diameters appear at outlet 1, it indicates that the equipment has experienced abnormal wear and tear. Immediate maintenance and replacement of components are necessary to prevent major accidents. The secondary sorting mainly targets particles smaller than 50 microns, which are the abrasive particles resulting from the early wear of the equipment. It enables precise sorting of these particles and can be used to study the wear patterns of different equipment during their operational periods, thereby supporting the formulation of more scientific equipment maintenance and repair plans. Furthermore, using permanent magnets directly as the external magnetic source for the differential selector has certain limitations regarding the magnitude of the magnetic field gradient. Later, high magnetic permeability soft magnetic materials can be placed between the permanent magnet and the channel. When the soft magnetic materials are magnetized by the permanent magnet, an induced magnetic field will be generated. By designing different shapes of soft magnetic materials, the magnetic field in the separation area is made to have a higher magnetic gradient, thereby increasing the resolution of the separator and achieving a narrower particle size distribution in the abrasive sample.

This paper theoretically analyzes the mechanism principle of using magnetic separation to sort ferromagnetic metal abrasive particles in the oil through the design of the chip structure, as well as the study of important parameters such as the position of the external magnetic source, the flow rate of the sample, the ratio of the sheath fluid to the sample flow rate, etc. Finally, a two-stage sorting oil particle separation chip was designed. The chip was fabricated using soft lithography technology, and an experimental platform was set up to verify the sorting effect of the chip through experiments. The simulation and experimental results show that the primary sorting can effectively separate large particles larger than 50 µm, and the secondary sorting can achieve fine separation of particles smaller than 50 µm. This research has expanded the application of microfluidic technology in the field of mechanical wear and at the same time has provided a new approach for the rapid detection of oil contamination degree.

## Figures and Tables

**Table 1:** The sorting efficiency and purity of the chips.

Outlets Name	Particle Diameter	Sorting Efficiency	Purity
Outlet 1	≥50 µm	95.37%	85.55%
Outlet 2	30–50 µm	80.13%	88.31%
Outlet 3	10–30 µm	87.55%	93.05%
Outlet 4	1–10 µm	93.05%	91.83%

## Data Availability

The original contributions presented in this study are included in the article. Further inquiries can be directed to the corresponding author.

## References

[1] Han W., Mu X., Liu Y., Wang X., Li W., Bai C., Zhang H. (2023). A Critical Review of On-Line Oil Wear Debris Particle Detection Sensors. J. Mar. Sci. Eng..

[2] Kumar M., Shankar M., Mohan M. (2013). Advancement and current status of wear debris analysis for machine condition monitoring: A review. Ind. Lubr. Tribol..

[3] Vamsi I., Sabareesh G., Penumakala P. (2019). Comparison of condition monitoring techniques in assessing fault severity for a wind turbine gearbox under non-stationary loading. Mech. Syst. Signal Process..

[4] Han W., Li W., Zhang H. (2024). Insight into mixing performance of bionic fractal baffle micromixers based on Murray’s Law. Int. Commun. Heat Mass..

[5] Krogsoe K., Henneberg M., Eriksen R. (2018). Model of a light extinction sensor for assessing wear particle distribution in a lubricated oil system. Sensors.

[6] Xu C., Zhang P., Wang H., Li Y., Lv C. (2015). Ultrasonic echo waveshape features extraction based on QPSO-matching pursuit for online wear debris discrimination. Mech. Syst. Signal Process..

[7] Sun J., Wang L., Li J., Li F., Li J., Lu H. (2021). Online oil debris monitoring of rotating machinery: A detailed review of more than three decades. Mech. Syst. Signal Process..

[8] Du L., Zhe J., Carletta J., Veillette R., Choy F. (2010). Real-time monitoring of wear debris in lubrication oil using a microfluidic inductive coulter counting device. Microfluid. Nanofluidics.

[9] Murali S., Jagtiani A.V., Xia X., Carletta J., Zhe J. (2009). A microfluidic coulter counting device for metal wear detection in lubrication oil. Rev. Sci. Instrum..

[10] Ren Y., Li W., Zhao G., Feng Z. (2018). Inductive debris sensor using one energizing coil with multiple sensing coils for sensitivity improvement and high throughput. Tribol. Int..

[11] Zhu X., Du L., Zhe J. (2017). A 3 × 3 wear debris sensor array for real time lubricant oil conditioning monitoring using synchronized sampling. Mech. Syst. Signal Process..

[12] Yan R., Gao R. (2024). Complexity as a measure for machine health evaluation. IEEE Trans. Instrum. Meas..

[13] Myshkin N., Markova L., Semenyuk M., Kong H., Han H.-G., Yoon E.-S. (2003). Wear monitoring based on the analysis of lubricant contamination by optical ferroanalyzer. Wear.

[14] Vähäoja P., Välimäki I., Heino K., Perämäki P., Kuokkanen T. (2003). Determination of wear metals in lubrication oils:: A comparison study of ICP-OES and FAAS. Anal. Sci..

[15] Neves A., Cesar C. (2019). Analytical calculation of optical forces on spherical particles in optical tweezers: Tutorial. J. Opt. Soc. Am. B.

[16] Waheed W., Sharaf O.Z., Alazzam A., Abu-Nada E. (2021). Dielectrophoresis-field flow fractionation for separation of particles: A critical review. J. Chromatogr. A.

[17] Li P., Huang T. (2019). Applications of Acoustofluidics in Bioanalytical Chemistry. Anal. Chem..

[18] Surendran A., Zhou R., Lin Y. (2021). Microfluidic Devices for Magnetic Separation of Biological Particles: A Review. J. Med. Devices.

[19] Han W., Wang X., Liu Y., Bai C., Li W., Zhang H. (2024). A Perspective Review of Droplets and Bubbles Formation in Microfluidics. Microgravity Sci. Technol..

[20] Han W., Wang X., Li W., Zheng Y., Liu B., Zhang H. (2024). Numerical simulation study of bubble breakup mechanism in microchannels with V-shaped obstacle. Chem. Eng. Process.

[21] Yang H., Li G., Wang J., Hou Q., Pan H., Quan J., Chen Y., Xu J., Liu Y., Liu H. (2024). Based microfluidic chip for sweat volume, ECG and EMG monitoring. Sens. Actuators A Phys..

[22] Wu L., Liu X., Zhang Y., Yang Z., Chen L., Zong S., Li J., Cui Y., Wang Z. (2024). A hand-powered SERS-microfluidic chip for circulating tumor DNA detection from whole blood. Sens. Actuators B Chem..

[23] Liu H., Liu Y., Yu X., Huang X., Zhang J., Chen Z., Xu J. (2024). A novel bubble-based microreactor for enhanced mass transfer dynamics toward efficient electrocatalytic nitrogen reduction. Small.

[24] Wu Z., Yang H., Xu H., Dai W., Xu L., Du H., Zhang D. (2024). A review on the development and application of microfluidic concentration gradient generators. Phys. Fluids.

[25] Chen X., Chen X. (2024). A novel electrophoretic assisted hydrophobic microdevice for enhancing blood cell sorting: Design and numerical simulation. Anal. Methods.

[26] Jing D., Qi P. (2024). The Optimal Branch Width Convergence Ratio to Maximize the Transport Efficiency of the Combined Electroosmotic and Pressure-Driven Flow within a Fractal Tree-like Convergent Microchannel. Fractal Fract..

[27] Zhou R., Yang Q., Bai F., Werner J.A., Shi H., Ma Y., Wang C. (2016). Fabrication and integration of microscale permanent magnets for particle separation in microfluidics. Microfluid. Nanofluid..

[28] Del Giudice F., Madadi H., Villone M.M., D’AVino G., Cusano A.M., Vecchione R., Ventre M., Maffettone P.L., Netti P.A. (2015). Magnetophoresis ‘meets’ viscoelasticity: Deterministic separation of magnetic particles in a modular microfluidic device. Lab Chip.

[29] Zhang J., Hou S., Cheng Q., Wang Y., Zang W., Duan J., Zhang B. (2024). Design of a high-throughput integrated microfluidic chip combining micromixing and particle sorting functions. Phys. Scr..

[30] Dang Y., Zhang Q., Hu S., Ou Z. (2024). A microparticle manipulation method facilitated via microfluidic chip based on swirling flow topology design and its application in sorting. J. Ind. Eng. Chem..

[31] Zhang Y., Zhang T., Zhang X., Cheng J., Zhang S. (2024). Label-Free Continuous Cell Sorting Using Optofluidic Chip. Micromachines.

[32] Hettiarachchi S., Ouyang L., Cha H., Hansen H.H.W.B., An H., Nguyen N.-T., Zhang J. (2024). Viscoelastic microfluidics for enhanced separation resolution of submicron particles and extracellular vesicles. Nanoscale.

